# Examining the utility of a photorealistic virtual ear in otologic education

**DOI:** 10.1186/s40463-022-00614-5

**Published:** 2023-02-22

**Authors:** Dongho Shin, Arthur V. Batista, Christopher M. Bell, Ella R. M. Koonar, Joseph M. Chen, Sonny Chan, Joseph C. Dort, Justin T. Lui

**Affiliations:** 1 Department of Otolaryngology–Head and Neck Surgery, University of Toronto, Toronto, Canada; 2 Ohlson Research Initiative, Arnie Charbonneau Cancer Institute, University of Calgary, Calgary, Canada; 3 Section of Otolaryngology–Head and Neck Surgery, Department of Surgery, Cumming School of Medicine, University of Calgary, Calgary, Canada; 4 Cumming School of Medicine, Faculty of Medicine, University of Calgary, Calgary, Canada; 5 Department of Computer Sciences, University of Calgary, Calgary, AB Canada

**Keywords:** Virtual reality simulator, Virtual reality, Otology, Anatomical models, Middle ear, Photorealism

## Abstract

**Background:**

Otolaryngology–head and neck surgical (OHNS) trainees’ operating exposure is supplemented by a combination of didactic teaching, textbook reading, and cadaveric dissections. Conventional teaching, however, may not adequately equip trainees with an understanding of complex visuospatial relationships of the middle ear. Both face and content validation were assessed of a novel three-dimensional (3D) photorealistic virtual ear simulation tool underwent face and content validation as an educational tool for OHNS trainees.

**Methods:**

A three-dimensional mesh reconstruction of open access imaging was generated using geometric modeling, which underwent global illumination, subsurface scattering, and texturing to create photorealistic virtual reality (VR) ear models were created from open access imaging and comiled into a educational platform. This was compiled into an educational VR platform which was explored to validate the face and content validity questionnaires in a prospective manner. OHNS post-graduate trainees were recruited from University of Toronto and University of Calgary OHNS programs. Participation was on a voluntary basis.

**Results:**

Total of 23 OHNS post-graduate trainees from the two universities were included in this study. The mean comfort level of otologic anatomy was rated 4.8 (± 2.2) out of 10. Senior residents possessed more otologic surgical experience (*P* < 0.001) and higher average comfort when compared to junior residents [6.7 (± 0.7) vs. 3.6 (± 1.9); *P* = 0.001]. Face and content validities were achieved in all respective domains with no significant difference between the two groups. Overall, respondents believed OtoVIS was a useful tool to learn otologic anatomy with a median score of 10.0 (8.3–10.0) and strongly agreed that OtoVIS should be added to OHNS training with a score of 10.0 (9.3–10.0).

**Conclusions:**

OtoVIS achieved both face and content validity as a photorealistic VR otologic simulator for teaching otologic anatomy in the postgraduate setting. As an immersive learning tool, it may supplement trainees’ understanding and residents endorsed its use.

**Graphical Abstract:**

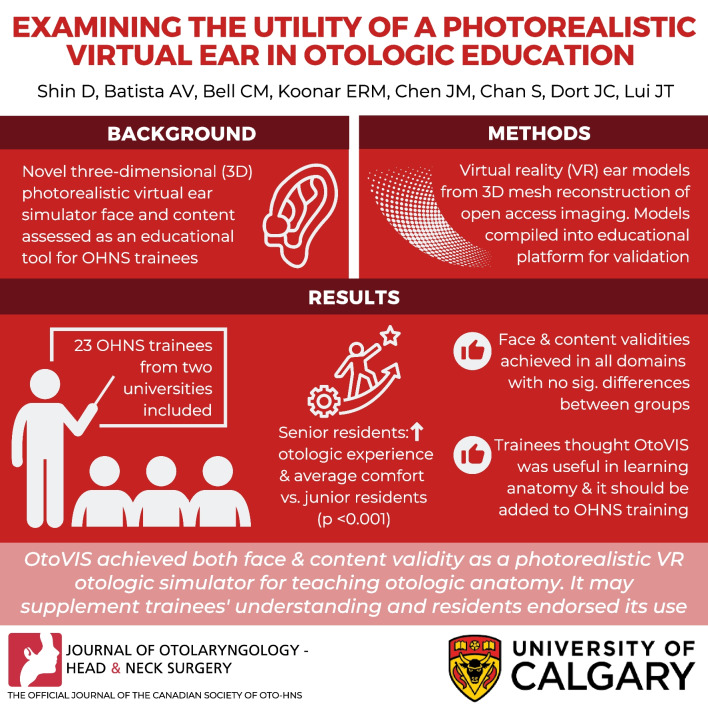

## Background

Middle ear anatomy is complex and a fundamental understanding of its visuospatial relationships is imperative for performing middle ear procedures [1]. Compounded by the need for microscopic or endoscopic magnification, the middle ear cleft is not easily accessible nor easily studied in the cadaveric setting [1]. Given these challenges, educational tools including paper models to three-dimensional computer models have been explored for middle ear teaching [2, 3]; however, shortcomings of the existing technologies include lack of a realistic appearance, including the retro- and hypotympanic spaces, which are poorly understood [1, 4].

**Fig. 1:**
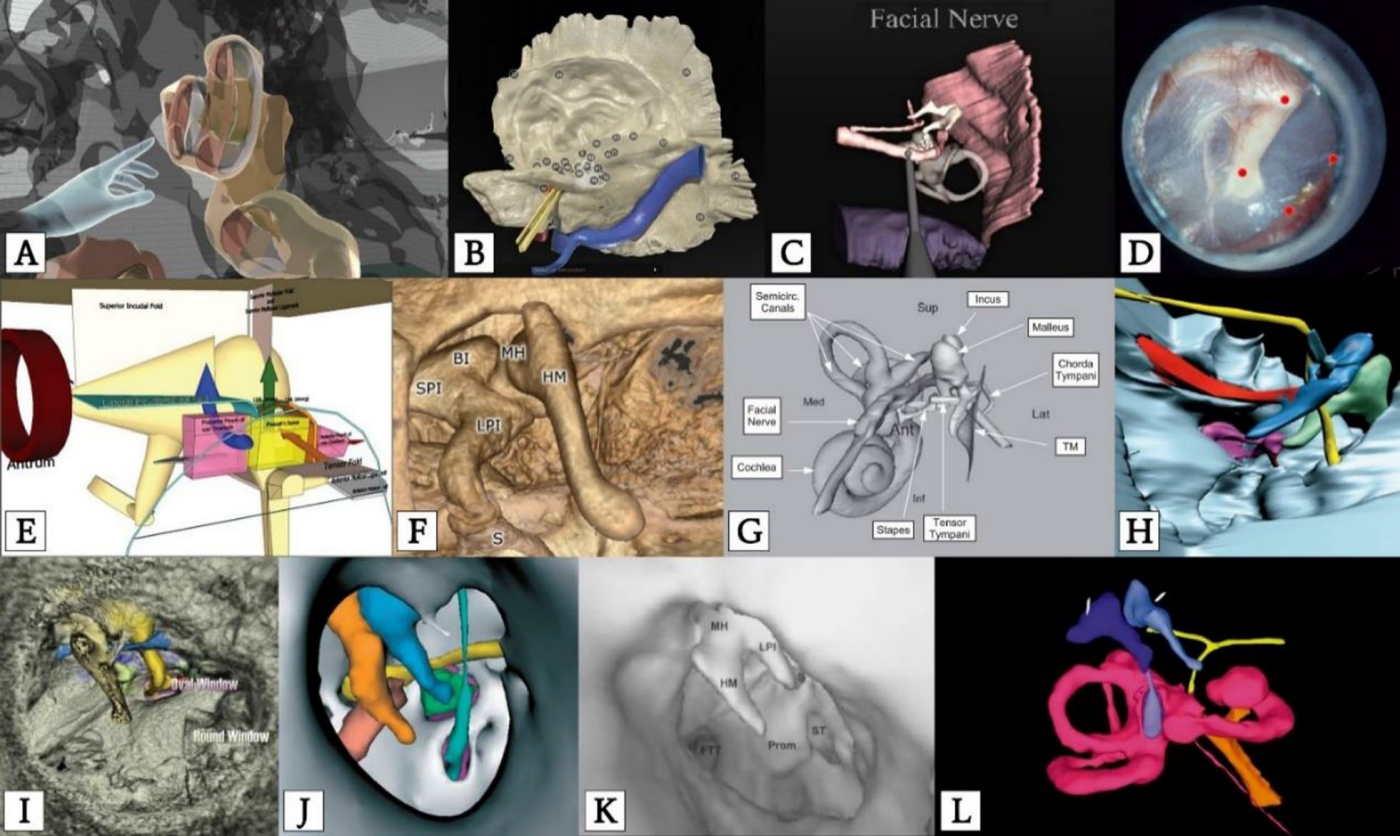
Existing ear simulators. Image adapted from Batista [19]. **A** Adams et al. [36]; **B** Hendricks et al. [34]; **C** Wijewickrema et al. [35]; **D** Samra et al. [33]; **E** Ng et al. [32]; **F** Lee et al. [42]; **G** Nicholson et al. [41]; **H** Dai et al. [43]; **I** Folowosele et al. [40]; **J** Rodt et al. [39]; **K** Neri et al. [38]; **L** Seemann et al. [37]

With more affordable computing power and advancements in virtual reality (VR) technology, the surgical training landscape is experiencing significant changes to fill the gaps in anatomical knowledge [5–9]; furthermore, commercially-available devices, such as the Oculus (Facebook Technologies LLC., Menlo Park, CA) VR headsets, have made it possible for trainees to engage in VR surgeries anywhere and anytime [10]. As such, VR training has garnered significant interest in otologic surgery involving the middle ear and temporal bone for numerous reasons [11]. From complex anatomy to the high degree of inter-patient variability, otologic anatomical conceptualization is challenging for many novice surgeons. Additionally, narrowed access into the middle ear via the external auditory canal further limits middle ear visibility in non-endoscopic surgeries. Although various otologic VR programs exist [11–15], the widespread use of computerized tomography (CT) datasets reduces visual realism of soft tissue and bone [16]. There continues to be a paucity in the Otolaryngology–Head & Neck Surgery (OHNS) programs of a full immersive experience using a head-mounted display [17]. The void of highly realistic and immersive outer and middle ear simulators makes it difficult to conceptualize current available VR models into real-life practice (Fig. 1).

This study aimed to bridge the gap between VR and realistic anatomy to improve the existing VR educational tools available in OHNS programs. Photorealistic digital artistry was employed to create an immersive and realistic environment of the external and middle ears to illustrate the complex three-dimensional area of the body. This was then compiled into a VR educational tool for OHNS trainees whereby face and content validity were assessed [18]. In OtoVIS’ context, face and content validity signify anatomic realism, and its appropriateness as a teaching modality, respectively. Measuring OtoVIS’ face and content validity is the first step into creating an immersive VR tool that can be incorporated into OHNS surgical training.

## Methods

**Fig. 2:**
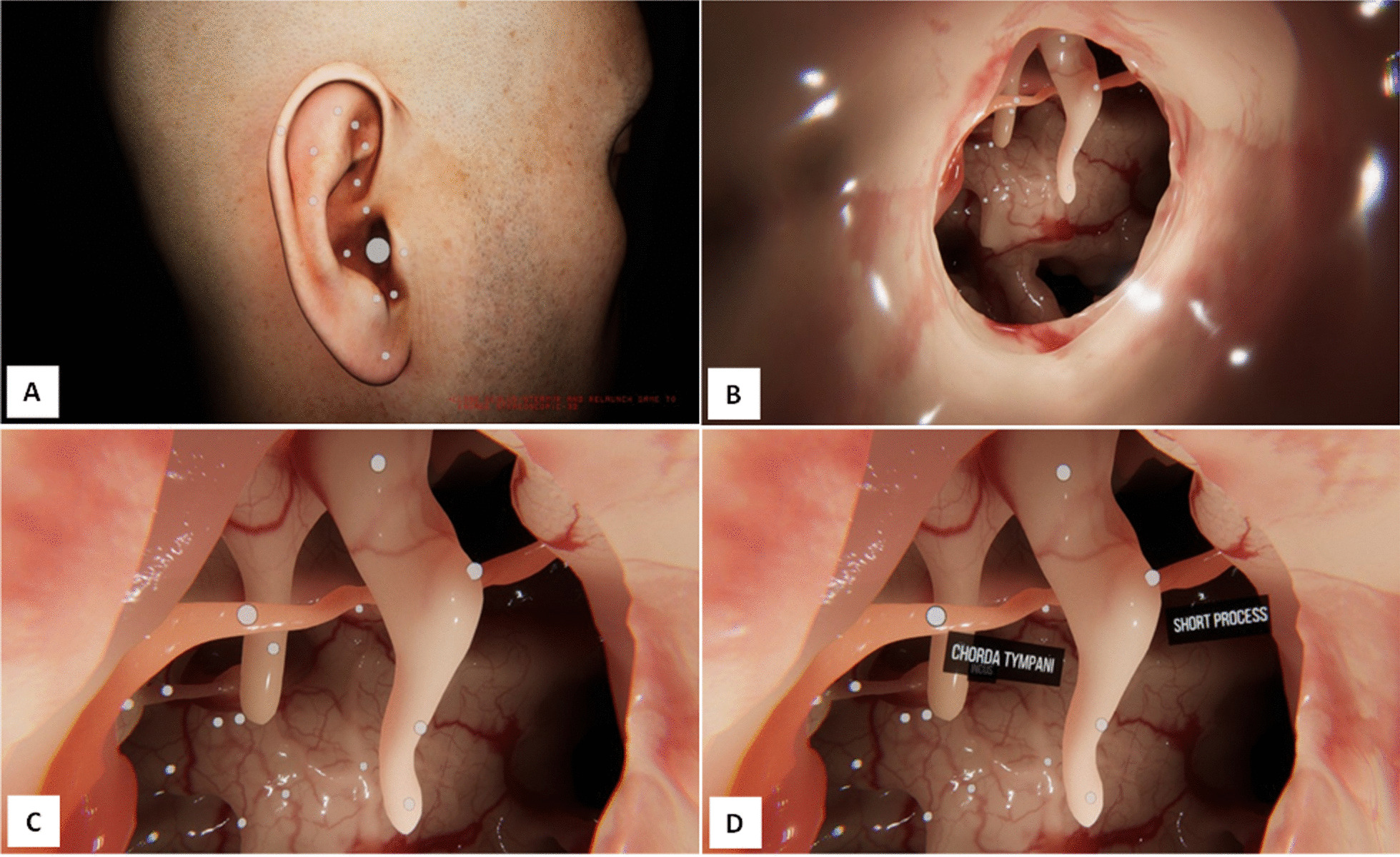
OtoVIS application images. Images were taken from OtoVIS during it simulation. External ear (**A**); external auditory canal and transcanal view of the middle ear (**B**); middle ear space and its anatomical structures (**C**); middle ear space with labels turned on (**D**)

Following approval by both institutional research ethics boards (Conjoint Health Research Ethics Board, 20–0360; Sunnybrook Research Ethics Board, SUN 2994) OHNS trainees from two postgraduate programs were recruited into the study from July 2020 to February 2021. Each participant received standardized introductions on the Oculus Rift S headset and to the OtoVIS application. Following this, participants were encouraged to explore the external ear, external auditory canal, and the middle ear independently (Fig. 2A–C). Different anatomical landmarks could be toggled on and off throughout the simulation (Fig. 2D).

### OtoVIS technology

The OpenEar library is comprised of eight high-fidelity, 3D models of the human temporal bone and served as the basis of OtoVIS [19]. The novel amalgamation of imaging from ionizing radiation and micro-slicing allowed for heightened detail of delicate soft tissue structures [16]. The dataset of specimen “zeta” was used as the model prototype in OtoVIS. The 3D mesh reconstruction of zeta reduced the number of polygons to improve VR performance, using a geometric modeling software called Blender 3D version 2.83 (Blender Foundation, Amsterdam, Netherlands). Additional anatomical structures, such as the stapedial tendon, tensor tympani tendon, Jacobson’s nerve, pyramidal eminence, and chorda tympani, were manually modelled into OtoVIS using endoscopic photos and expert surgeons’ feedback. Finally, the external ear and the human head model was adapted from an online store, 3D Scan Store (Ten24 Media LTD., Sheffield, UK).

The Unity platform (Unity Software Inc., San Francisco, CA) and its high-definition rendering pipeline technology was utilized to allow physical settings in OtoVIS to behave as they would in the real world. This allowed for global illumination (simulating realistic lighting in a 3D environment creating reflections, shadows, and refractions), subsurface scattering (the way light is absorbed and scattered on a translucent material), and texturing to be applied to OtoVIS. Texture mapping of blood vessels and nerves, glossy surfaces, translucency, color variations and shadows were referenced from Pollak’s endoscopic view of the middle ear [20]. The depth of field, chromatic aberrations, film grain, motion blur, lens distortion, tone-mapping, and white balance were used to enhance realism of OtoVIS. Lastly, Oculus Integration Library (Facebook Technologies LLC., Menlo Park, CA) was used to set up the functionalities of the VR environment.

### Face and content validity

To assess face and content validity, post-simulation data were collected from participants using self-assessment surveys. The residents completed a questionnaire with a Likert scale ranging from 0 (strongly disagree) to 10 (strongly agree). The questions were organized into five face and five content domains, respectively. Face validity domains included questions regarding the satisfaction of the 3D representation of the external and middle ear structures. Content validity domains covered questionnaires about OtoVIS’ usefulness as an educational tool. Responses were reported as the median score with a 25th–75th percentile. A score of 8 or higher was used as the benchmark for validating each domain. Demographic data were collected including the post-graduate year (PGY), estimated number of participated middle ear surgeries, comfort with middle ear anatomy, and preferred learning resource. All responses were anonymized for confidentiality.

Statistical analysis was performed with SPSS statistical package, version 26 (IBM Corp., Armonk, NY). Both Fisher’s exact and Chi-square testing were used for demographic data. The Mann–Whitney U test was used to compare the responses of residents when separating junior from senior trainees.

### Thematic analysis

Free-text comments about advantages and disadvantages of OtoVIS were also collected after the Likert scale questionnaire in an open-ended manner. These data were explored in a qualitative fashion using thematic analysis. The free texts were coded into subcategories to define themes.

## Results

### Demographics

Twenty-three OHNS residents were included in the study (Table 1). All PGY-levels were represented: 4 PGY-1 (17%), 7 PGY-2 (30%), 3 PGY-3 (13%), 6 PGY-4 (26%) and 3 PGY-5 (13%). The residents were grouped into junior (PGY 1–3; n = 14) and senior (PGY 4–5, n = 9) residents for comparison. Out of the 23 participants, 11 were male (48%) and 12 were female (52%). No residents were excluded from the analysis.

**Table 1:** Demographic information. Post-graduate year (PGY); Standard deviation (SD)

	Total	Junior Residents (n = 14)	Senior Residents (n = 9)	*p*-value
Sex				0.265
Male	11 (48%)	8 (57%)	3 (33%)	
Female	12 (52%)	6 (43%)	6 (67%)	
PGY-level				–
1	4 (17%)	-	-	
2	7 (30%)	-	-	
3	3 (13%)	-	-	
4	6 (26%)	-	-	
5	3 (13%)	-	-	
Comfort with middle ear anatomy (mean ± SD)	4.8 ± 2.2	3.6 ± 1.9	6.7 ± 0.7	< 0.001*
Estimated # of middle ear surgeries				< 0.001*
0–5	11 (48%)	11 (79%)	0 (0%)	
6–10	4 (17%)	2 (14%)	2 (22%)	
11–30	3 (13%)	1 (7%)	2 (22%)	
> 30	5 (22%)	0 (0%)	5 (56%)	
Preferred learning resource:		–		–
Textbooks/anatomy atlases	22 (96%)	13 (93%)	9 (100%)	
Lecture material	19 (83%)	11 (79%)	8 (89%)	
Intraoperative experience	14 (61%)	6 (43%)	8 (89%)	

The majority of participants were involved in ten or less middle ear surgeries (48% had 0–5 middle ear surgeries, and 17% had 6–10 middle ear surgeries). Only 35% of residents had more than ten middle ear surgical experiences (Table 1). Senior residents possessed more otologic surgical experience when compared to junior residents (*p* < 0.001). 79% of junior residents participated in five or less middle ear surgeries while 56% of senior residents had more than 30.

Residents learned anatomy mostly from anatomical atlases and lecture materials (96%, 83%, respectively). Only 61% of residents relied on intraoperative experiences to guide their middle ear understanding. Prior to using OtoVIS, the mean comfort level of middle ear anatomy rated from 0 to 10 was 4.8 ± 2.2 (Table 1). Senior residents also had significantly higher average comfort of middle ear anatomy than junior residents (6.7 ± 0.7 vs. 3.6 ± 1.9; *p* = 0.001). No resident rated their comfort as 10 out of 10.

### Face and content validity

**Fig. 3:**
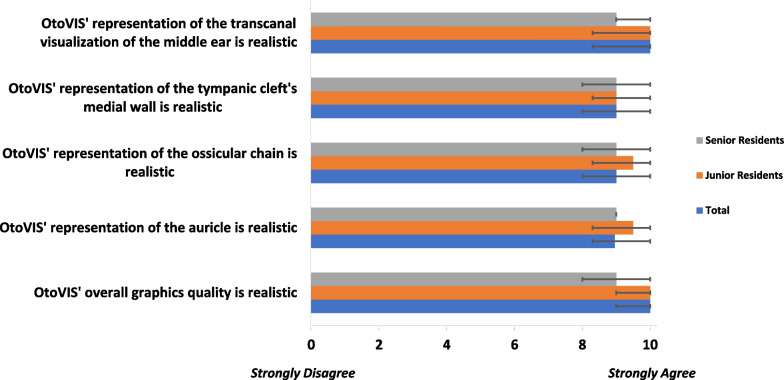
Face validation assessment of OtoVIS

**Fig. 4:**
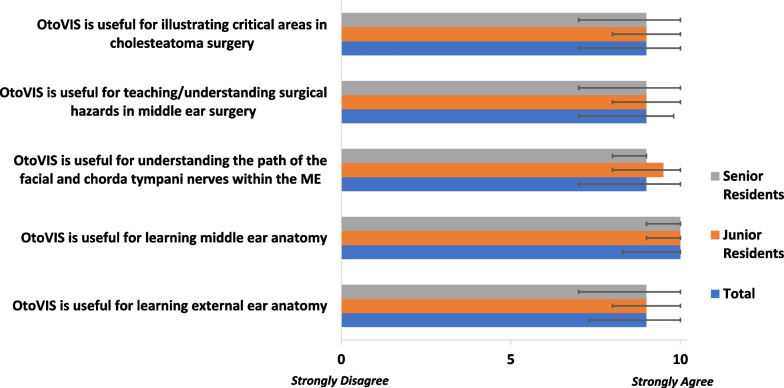
Content validation assessment of OtoVIS

The five domains of face validity realism were assessed including the overall graphical rendering, the realism of transcanal visualization of the middle ear, and specific representation of various anatomical structures such as the pinna, ossicular chain, and the medial wall of the tympanic cleft. The five domains of content validity (usefulness and appropriateness for teaching module) included: learning external ear anatomy, understanding middle ear anatomical orientation, understanding the path of the facial and chorda tympani nerves, comprehending surgical hazards in middle ear surgery, and illustrating critical areas in cholesteatoma surgery. OtoVIS was considered realistic in all five face validity domains and useful in all five content validity domains (Figs. 3, 4). There were no significant differences in the responses to face and content validities between the junior and senior residents (Figs. 3, 4).

### Global rating

Respondents were queried about OtoVIS’ ease of use and its suitability for OHNS training (Fig. 4). Moreover, respondents’ willingness to recommend to colleagues, their ability to apply obtained knowledge to clinical practice, and potential utility in patient education were also examined (Fig. 4). Respondents strongly agreed that OtoVIS should be added to OHNS residency training (10.0; 9.3–10.0) and would recommend OtoVIS to a colleague (10.0; 8.3–10.0). Trainees felt OtoVIS would be applicable to clinical practice (10.0; 9.0–10.0) and useful for patient education (9.0; 7.0–10.0). There were no significant differences in the responses of junior and senior residents (Fig. 4).

### Open-ended feedback

The results from the free text comments were summarized in Table 2. The two main themes that were analyzed was “educational tool” (defined as: how OtoVIS has the potential to be a good educational tool) and “challenges” (defined as: potential pitfalls of OtoVIS). Participants identified a safe learning environment, great visual representation, and interactive nature of OtoVIS as its strengths as an educational tool. They also recognized cost, technical difficulties and the user interface to be a potential challenge for OtoVIS.

**Table 2:** Description of thematic analysis of general comments

Educational tool	How OtoVIS has the potential to be a good educational tool	
Subcategories	Description	Significant statement examples
Learning environment	Relationship of OtoVIS and its contribution to the learning environment	"Low risk environment for learning difficult anatomy and surgical techniques"
Visual representation	The visual accuracy and representation of the anatomy	"Best representation of middle ear anatomy I have every seen"
Interaction	Interactive component of OtoVIS	"Fun interactive, residents/staff would likely be interested and motivated to use this technology for learning"

Challenges	Potential pitfalls of OtoVIS	
Subcategories	Description	Significant statement examples
Cost & equipment	The amount of money required to set up OtoVIS	"Set up (buying and setting up VR equipment)—that would be easily solved by department purchasing this and incorporating in temporal bone labs"
Technical difficulties	Technical difficulties ran into during simulation	"Bit buggy for now. Required restart during my run"
User interface	Participants experience with user interface	"Requires some time to acclimate to the software and hand manipulation to control."

## Discussion

Understanding middle ear anatomy and its complex three-dimensional nature is essential for otologic procedures [11]; however, knowledge translation of spatial relationships from two-dimensional images and the lack of operative exposure challenges a trainee’s ability to learn middle ear anatomy [21]. Although 95.7% of respondents used textbooks and atlases for anatomical understanding, operative exposure in the later years of postgraduate training may explain the significant improvement in comfort levels with middle ear anatomy between junior (3.6 ± 1.9) and senior trainees (6.7 ± 0.7). Often, available resources lack realism to accurately depict anatomical relationships especially when operative experience is limited [22]. This is especially true in middle ear surgeries where the three-dimensional spatial relationships are conceptually difficult, and visualization of the operative field is limited for trainees [10, 23].

OHNS has pioneered the use of digital graphics to improve trainees’ knowledge and skill acquisition in numerous virtual and augmented reality platforms [8, 11, 12, 14, 15, 24]. VR’s unique ability to immerse trainees in an interactive environment provides numerous possibilities to enhance learning [8, 11–13, 25]. VR promotes active learning by increasing physical interaction and exploration of the subject matter which has been shown to improve learning efficiency and retention [26–29]. The full immersion aspect of VR aids in information recall and motivates students for higher interests [30, 31]. Unfortunately, studies or applications that focus on an immersive VR representation of a detailed, in-vivo middle ear soft tissue and bones seen through the endoscopic view are lacking in the last five years [32–36]. The current technological paradigm of immersive VR is not being fully utilized in the educational OHNS surgical training. Previous investigations that presented virtual endoscopic visualization of the middle ear used outdated technologies and lacked photorealistic graphics quality [37–43] (Fig. 2). Although these studies showed the importance of endoscopic view in conceptualizing a challenging anatomy, the missing technicalities of realistic color information, textures and reflections prevented the creation of a life-like educational model; therefore, OtoVIS was generated to create a photorealistic, texture-rich outer and middle ear rendering to add to a trainee’s armamentarium to overcome the learning curve.

By creating textures with dynamic lighting and shadowing effects, photorealism blends VR visuals to look strikingly realistic [19]. This is the foundation of the OtoVIS platform, which fills a void of realistic three-dimensional middle ear reconstructions resembling live, in-vivo anatomy. Respondents agreed OtoVIS’ visual rendering of the pinna, ossicular chain, medial wall of the tympanic cleft were highly accurate (Fig. 3). Additionally, users felt the ability to explore a photorealistic middle ear cleft proved usefulness in understanding the paths of the facial and chorda tympani nerves in the middle ear (9.0, 7.0–10.0). Furthermore, OtoVIS’ utility in depicting complex areas such as the sinus tympani provide opportunities to understand how anatomy can influence cholesteatoma development or highlight the areas critical to successful surgery (9.0, 7.0–10.0). This detailed level of three-dimensional spatial anatomy would only be explored from intra-operative experience prior to the creation of OtoVIS. This was seen from the thematic analysis of participants’ open-ended feedback. OtoVIS provided “low risk environment for learning difficult anatomy and surgical techniques” with the “best representation of middle ear anatomy” which was “fun and interactive”. “Residents/staff would likely be interested and motivated to use this technology for learning”.

**Fig. 5:**
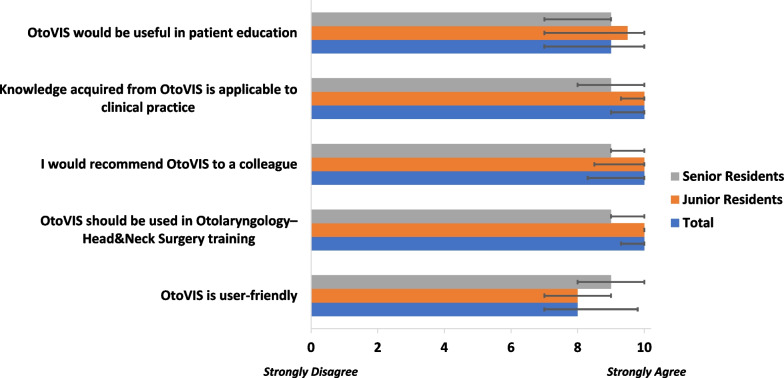
Global rating assessment of OtoVIS

Face and content validities are necessary in evaluating the utility of surgical simulation tools [18]. All domains met or exceeded the threshold of validity confirming OtoVIS as a realistic VR simulation of the middle ear space and would be an appropriate asset to be used as an educational tool for OHNS surgical training (Fig. 4). When stratifying by trainee experience, responses were positive in both groups (Figs. 3, 4, 5). Overall, trainees strongly agreed that OtoVIS should be used in OHNS training (10.0, 9.3–10.0) and they would recommend it to a colleague (9.0, 8.3–10.0). Additionally, OtoVIS was thought to be useful for clinical practice (9.0, 9.0–10.0) and beneficial for patient education (9.0, 7.0–10.0).

### Future steps

OtoVIS’ ability to translate users’ heightened spatial understanding into meaningful understanding of the mechanics of the middle ear or even how diseases such as cholesteatoma may spread requires further evaluation to confirm these encouraging results [18]. Its widespread dissemination as a platform for other regions of the head and neck may position it as a complementary tool in a trainee’s armamentarium.

The flexibility of OtoVIS and head-mounted VR makes it possible to combine other technologies to incorporate a hands-on surgical experience and develop surgical techniques. With the increasing popularity of endoscopic ear surgery, OtoVIS’ photorealism provides an immersive platform for future development of surgical rehearsal.

### Limitations of the study

Limitations of this study included the restriction of responses to trainees only. As well, the majority of participants were junior residents with minimal surgical experience. Experienced otologists were not included in this study, which could affect face validity. However, this investigation was aimed at comparing OtoVIS with the current self-guided format of post-graduate training. Moreover, the development of this simulator involved in the input of expert surgeons. Another challenge that was highlighted in the open-end responses was cost. OtoVIS requires several pieces of hardware including a headset paired with a dedicated consumer-grade computer with a moderate graphics processing unit. To supplement or even supplant current materials, adaptation to mobile devices is crucial in high-paced clinical training.

This represents the first study of OtoVIS and its appropriateness as a teaching modality. While face and content validities were assessed, this initial study was limited in measuring knowledge or skill acquisition which requires further investigation. Lastly, OtoVIS is designed from a highly detailed dataset with significant amount of photorealistic processing to generate one specimen. Without variability, trainees may become akin to believing the simulation is default anatomy. Therefore, continued efforts will include multiple specimens, pathologies, and anomalies.

## Conclusions

The unique application of photorealism into the outer and middle ear has not been previously endeavored. The heightened realism has enabled for a highly realistic, immersive interaction of trainees and the outer and middle ear. In addition to its utility in improving anatomical understanding, OtoVIS is positioned to dramatically change the traditional paradigm of learning in Otology. The results of this investigation although subjective, provide meaningful insight into the utility that this photorealistic anatomical simulator provides.

## Data Availability

The datasets used and/or analysed during the current study are available from the corresponding author on reasonable request.

## Abbreviations

OHNS: Otolaryngology–Head & Neck Surgery
VR: Virtual reality
CT: Computerized tomography
PGY: Post-graduate year
SD: Standard deviation
